# Ambipolar light-emitting organic single-crystal transistors with a grating resonator

**DOI:** 10.1038/srep10221

**Published:** 2015-05-11

**Authors:** Kenichi Maruyama, Kosuke Sawabe, Tomo Sakanoue, Jinpeng Li, Wataru Takahashi, Shu Hotta, Yoshihiro Iwasa, Taishi Takenobu

**Affiliations:** 1 Department of Applied Physics, Graduate School of Advanced Science and Engineering, Waseda University, Tokyo 169-8555, Japan; 2 Department of Physics, Graduate School of Science, Tohoku University, Sendai 980-8578, Japan; 3 Faculty of Materials Science and Engineering Kyoto Institute of Technology, Kyoto 606-8585, Japan; 4 Quantum-Phase Electronics Center and Department of Applied Physics, The University of Tokyo, Tokyo 113-8656, Japan, RIKEN Center for Emergent Matter Science (CEMS), Wako 351-0198, Japan

## Abstract

Electrically driven organic lasers are among the best lasing devices due to their rich variety of emission colors as well as other advantages, including printability, flexibility, and stretchability. However, electrically driven lasing in organic materials has not yet been demonstrated because of serious luminescent efficiency roll-off under high current density. Recently, we found that the organic ambipolar single-crystal transistor is an excellent candidate for lasing devices because it exhibits less efficient roll-off, high current density, and high luminescent efficiency. Although a single-mode resonator combined with light-emitting transistors (LETs) is necessary for electrically driven lasing devices, the fragility of organic crystals has strictly limited the fabrication of resonators, and LETs with optical cavities have never been fabricated until now. To achieve this goal, we improved the soft ultraviolet-nanoimprint lithography method and demonstrated electroluminescence from a single-crystal LET with a grating resonator, which is a crucial milestone for future organic lasers.

Organic semiconductors combine optoelectronic properties with simple fabrication methods and the ability to tune the chemical structure to yield desired features, making them attractive candidates as laser materials^1^,^2^. Optically pumped lasers constructed from a broad range of conjugated polymers^2^ and oligomers^3^,^4^,^5^ have been reported. However, electrically pumped organic lasers have not yet been demonstrated due to serious luminescent efficiency roll-off under high current density. In organic light-emitting diodes, the maximum current density that allows for a high emission efficiency is approximately 1–10 A/cm^2^,^6^,^7^ which is considerably lower than the estimated current density necessary for electrically driven lasing (>10 kA/cm^2^)^8^,^9^. Therefore, a novel device structure that enables high current densities and reduces efficiency roll-off is required.

Recently, an extremely high current density greater than 30 kA/cm^2^ was observed in ambipolar single-crystal light-emitting transistors (SCLETs) of α,ω-bis(biphenylyl)terthiophene (BP3T, Fig. 1a)^10^ due to their excellent transfer properties. In this report, the current density at the roll-off point was improved to 1 kA/cm^2^,^10^ resulting from a reduced exciton quenching effect in the single-crystal transistor configuration. These results indicate that ambipolar SCLETs are promising devices for future organic lasers, and reducing the lasing threshold below 1 kA/cm^2^ is the last remaining issue to be solved.

An effective method for lowering the necessary exciton density for lasing is the application of optical resonators^2^. Although it is difficult to fabricate optical cavities with fragile organic single crystals, several attempts have been made. Examples include the Fabry-Pérot cavity^11^,^12^,^13^, microcavity^14^,^15^,^16^, and distributed feedback (DFB) resonator^17^,^18^,^19^,^20^,^21^. However, because of their relatively long cavity lengths (>2 μm), it is impossible for the Fabry-Pérot cavity and microcavity to demonstrate single-mode lasing, a property that is crucial for enhancing optical gain. In stark contrast to other cavities, the DFB structure is able to act as a single-mode resonator. However, because the sub-micrometer patterning of the organic single crystals that are necessary for the DFB cavity induces various problems, such as exciton-recombination centers and carrier traps, the fabrication of SCLETs with DFB structures is still a considerable challenge and a crucial milestone for the future development of organic lasers.

In this article, we demonstrate electroluminescence (EL) from SCLETs controlled by a sub-micrometer one-dimensional (1D) grating structure. BP3T single crystals were selected as the optical and electrical medium because of their excellent emission^22^, transport^23^, and solvent-tolerant properties^24^. We developed a soft UV-nanoimprint lithography (NIL) method^25^,^26^ and applied this technique to BP3T single crystals. We successfully observed modified EL from BP3T SCLETs with 1D grating structures. Additionally, the EL spectra that we obtained are well explained by Bragg diffraction and mode coupling resulting from the DFB system, enabling us to realize single-mode lasing.

## Results

### Fabrication of grating resonators on fragile organic single crystals

**Figure 1:**
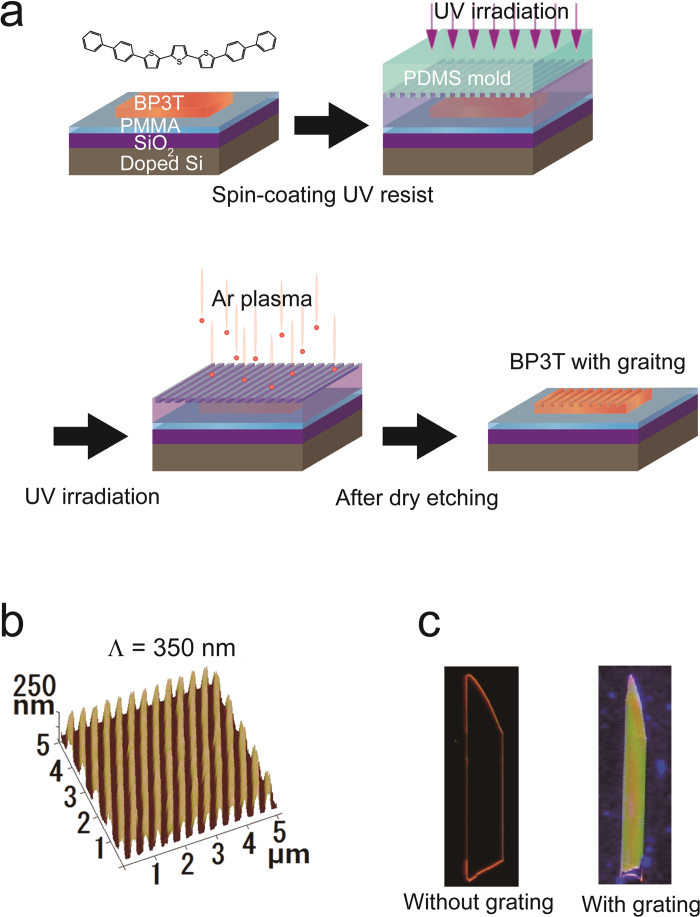
Fabrication of a sub-micrometre one-dimensional (1D) grating structure. (**a**) Molecular structure of α,ω-bis(biphenylyl)terthiophene (BP3T) and schematic fabrication steps for the soft ultraviolet (UV)-nanoimprint lithography (NIL) method. 1st step: BP3T single crystals were laminated onto a SiO_2_/Si substrate covered with poly(methyl methacrylate) (PMMA). A poly(dimethylsiloxane) (PDMS) mold with fine structure was also prepared. 2nd step: After spin coating UV-curable resist onto the BP3T crystal surface, the PDMS mold was pushed onto the resist film surface and irradiated with UV light, curing the resist film with the fine PDMS surface pattern. 3rd step: We completely etched the UV resist film with Ar plasma to copy the resist surface’s fine structure to the BP3T crystal surface. (**b**) Atomic force microscopic (AFM) images of the BP3T single crystal illustrating the 1D grating structure (350-nm period). (**c**) Fluorescence micrographs of BP3T single crystals without and with 1D grating.

As an initial attempt to apply the UV-NIL method, we recently succeeded in fabricating a 1D grating structure on the single-crystal surface of 2,5-bis[4-(5’-phenylthiophen-2’-yl)phenyl]thiophene and 2,5-di((E)-styryl)thieno[3,2-b]thiophene^27^. In this work, we have further elaborated the UV-NIL technique by largely improving fabrication processes and appropriately choosing a robust material. To this end, we selected BP3T as the material because of its excellent carrier mobilities (hole mobility >1 cm^2^/Vs and electron mobility > 0.1 cm^2^/Vs)^23^ and internal photoluminescence (PL) efficiency (quantum efficiency ~80%)^22^. We also measured an external PL efficiency of BP3T, which was 32% in powder and 13% in single crystals. A particularly important material property for high-quality UV-NIL is solvent tolerance, as the UV-NIL method requires the spin coating of photoresist on the crystal surface (Fig. 1a). BP3T crystals dissolve sparingly in most organic solvents^24^, so an improvement in fabrication quality is expected.

The UV-NIL process is schematically shown in Fig. 1a and explained in the Methods and Supplementary Information S1. BP3T single crystals were grown by physical vapor deposition^23^,^28^ and laminated onto a SiO_2_/Si substrate covered with poly(methyl methacrylate), which enabled the fabrication of SCLETs by reducing the effect of electron traps on the SiO_2_ surface^29^. A poly(dimethylsiloxane) (PDMS) mold with a fine structure was also prepared. After spin coating UV-curable resist onto the BP3T crystal surface, we pushed the PDMS mold onto the resist film surface and irradiated the resist film with the fine PDMS surface pattern using UV light curing. We completely etched the UV resist film with Ar plasma to copy the resist surface’s fine structure to the BP3T crystal surface. To obtain a superior structure to that reported previously^27^, we optimized several parameters, including the PDMS peeling-off conditions, UV exposure time, dry etching power, and etching gas species (Supplementary Information S2).

The effectiveness of the modified method was examined by atomic force microscopy (AFM). We used Si substrates patterned by e-beam lithography and dry-etching techniques as master molds. We tested five different fine structures to judge the processing accuracy of our improved method: 1D grating patterns with periodicities of 1 μm, 350 nm, and 270 nm and two-dimensional (2D) periodic pillars and holes. As shown in Fig. 1b (and Supplementary Information S3), we successfully fabricated all of these patterns on BP3T crystal surfaces. Surprisingly, the qualities of the structures were better by far than those reported previously^27^, indicating the improved patternability of BP3T crystals due to their excellent solvent tolerance^24^ and the optimized UV-NIL method. To gain further macroscopic information, we took PL images of the crystal before and after etching (Fig. 1c). The PL of BP3T single crystals is strongly confined due to the crystal’s self-waveguide effect, resulting in light emission from the crystal’s fringe only^11^,^13^,^23^. This light confinement is due to the perfect alignment of molecules within the crystal. In BP3T thin crystals, the transition dipoles are perpendicular to crystal surface. Therefore, PL light reveals TM polarization^23^. However, the PL image clearly exhibited a different color emission from the crystal surface for the BP3T single crystals with 1D grating (350 nm period), suggesting luminescence control by the DFB system.

### Fabrication of ambipolar organic SCLETs with a grating resonator

**Figure 2:**
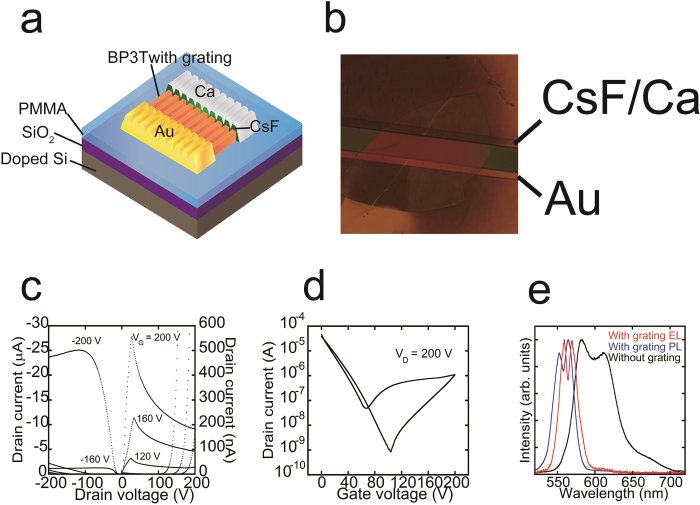
BP3T SCLETs with a DFB resonator. (**a**) Schematic structure of the BP3T SCLET with the 1D grating. (**b**) Optical micrograph of the fabricated SCLET with CsF/Ca and Au asymmetric electrodes. (**c**) Output characteristics of a BP3T single-crystal ambipolar device with 350-nm-period 1D grating at various applied gate voltages (*V*_*g*_). (**d**) Transfer characteristics of the device at *V*_D_ = 200 V. (**e**) An EL spectrum from a BP3T single-crystal SCLET during ambipolar operation with 350-nm period 1D grating (red line) and a PL spectrum from the same device (blue line). A PL spectrum from a BP3T single crystal without grating is also shown by the black line.

Following our success in crystal patterning, we fabricated SCLETs using BP3T single crystals with DFB cavities according to the method described by Sawabe *et al*^10^. The structure of the SCLET with 1D grating structure is shown in Fig. 2a,b. Au and Ca asymmetric electrodes were used to match the metal-electrode work function to the highest- and lowest-occupied molecular orbitals of BP3T^11^,^23^. Moreover, we used cesium fluoride (CsF) thin films (thickness ~0.6 nm) as an electron injection buffer layer^10^. The output and transfer characteristics of this device revealed clear ambipolar transport (Fig. 2c,d). The estimated hole and electron mobilities from the transfer curve were 2.1 and 0.014 cm^2^/Vs, respectively. We observed clear light emission during ambipolar transport, even though the electron mobility of this device was considerably smaller than typical BP3T single crystal transistors (>0.1 cm^2^/Vs)^23^. Because the electron mobility of etched crystals was extremely sensitive to UV exposure time, we optimized this parameter and realized detectable light intensities (Supplementary Information S4).

Similar to that of PL, the EL from BP3T SCLET with a 350-nm 1D grating structure was from surface emission (Supplementary Information S5). To further confirm the effect of the grating structure, we measured both PL and EL spectra from patterned BP3T SCLET and compared these spectra with PL from BP3T SCLET without any grating structure (Fig. 2e). The emission spectra from SCLET with the DFB system are narrower than those from non-patterned BP3T single crystals, confirming the successful control of luminescent properties by the 1D structure.

We fabricated 6 BP3T SCLETs with a 1D grating structure to investigate the device statistics (Supplementary Information S6). Four of the six devices revealed ambipolar transport, whereas we observed only p-type behavior for the other two transistors. In stark contrast to the similar hole mobilities among the six devices (1.1 - 2.1 cm^2^/Vs ), the electron mobilities that we obtained were scattering (3.5 × 10^−3^ – 1.4 × 10^−2^ cm^2^/Vs), suggesting again that the electron mobility of patterned crystals is easily affected by UV irradiation. Although two of the six transistors showed unipolar characteristics, we observed obvious light emission from all devices, and their wavelength was controlled by the DFB system. In particular, in the ambipolar BP3T SCLETs with a 1D grating structure, the light emitting zone was controlled by gate voltages, as reported in other ambipolar SCLETs (Supplementary Movie)^30^,^31^,^32^.

Moreover, we should compare the device performance of patterned BP3T SCLETs with BP3T SCLETs without etching (Supplementary Information S7). As we noted above, the hole mobilities of etched transistors (1.1 - 2.1 cm^2^/Vs) are close enough to those of normal transistors (0.2 - 1.64 cm^2^/Vs)^10^,^13^,^23^. However, the mobilities of electron transport in patterned BP3T SCLETs (3.5 × 10^−3^ – 1.4 × 10^−2^ cm^2^/Vs) are approximately one or two orders of magnitude smaller than those of reported BP3T SCLETs (0.17 – 0.2 cm^2^/Vs)^10^,^13^,^23^, indicating that there is still plenty room for improvement through optimizing the fabrication process and through the possibility of increasing the current density.

Interestingly, in Fig. 2e, both PL and EL spectra exhibited peak splitting in the SCLET with 1D grating. This splitting behavior might be explained by the Bragg scattering of propagated emission inside single crystals^33^ or by a photonic band gap^34^,^35^. It is very difficult to collect light emission that is exactly normal to the surface of crystals, and in the DFB system, the slight difference from the normal leads to peak splitting that is due to the Bragg scattering of propagated light. Moreover, if there is a non-negligible photonic band gap in the photonic crystal of the DFB system, peak splitting will be observed even in spectra from the correct direction. Therefore, we cannot determine the origin of the peak splitting in Fig. 2e without taking angular dependent measurements.

## Discussion

**Figure 3:**
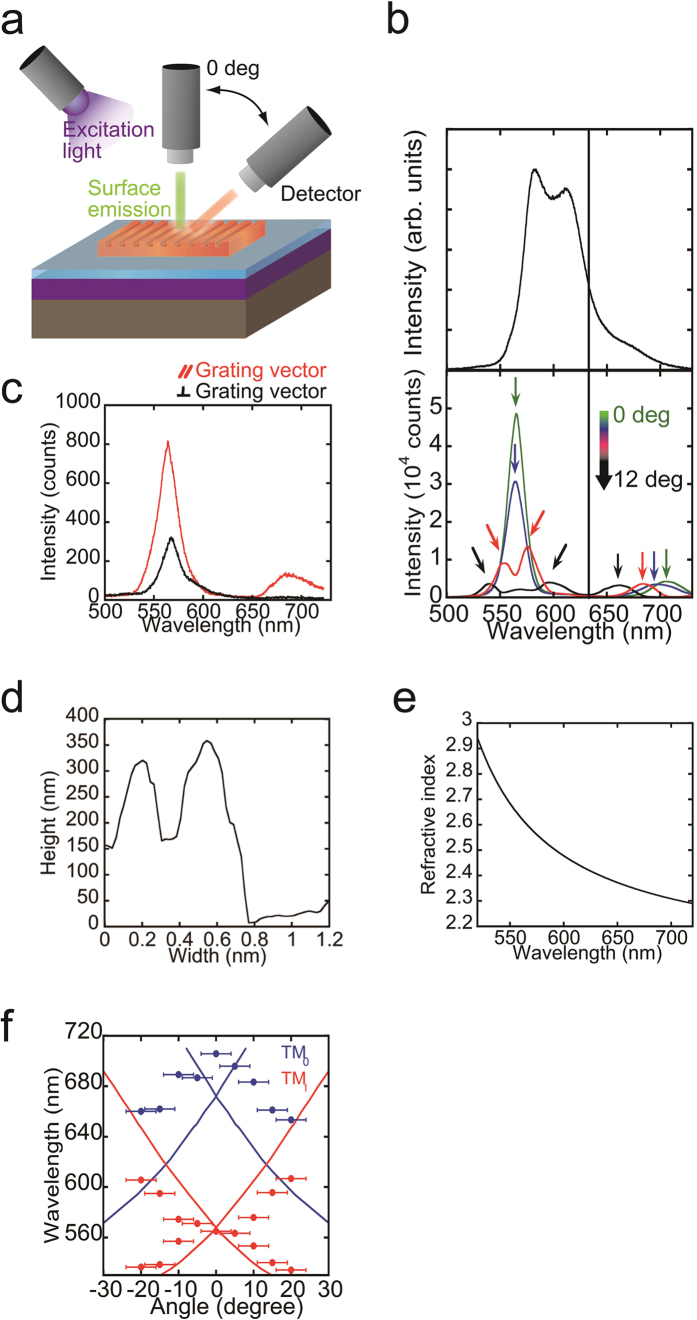
Optical properties of BP3T single crystals with a DFB resonator. (**a**) Schematic representation of angular-dependent PL measurements in BP3T single crystals with grating. (**b**) Top: a PL spectrum from a BP3T single crystal without a grating structure. Bottom: Angular dependence of PL spectra from BP3T single crystals with 350-nm 1D grating. (**c**) Polarized PL spectra from a BP3T single crystal with 350-nm 1D grating. Red and black correspond to polarization directions that are parallel and perpendicular, respectively, to the grating vector of the 1D grating. (**d**) Surface profile of a BP3T single crystal with a 350-nm-pitch grating structure. The height, depth, and grating period were determined to be approximately 363, 178, and 350 nm, respectively. (**e**) Refractive index dispersion of a BP3T single crystal. (**f**) Angular dependence of observed and predicted PL peak wavelength (Blue: TM_0_ mode, Red: TM_1_ mode).

To gain additional information about the peak splitting in the patterned BP3T crystal (Fig. 2e), we investigated the angular dependence of the PL spectra (Fig. 3a). As shown in Fig. 3b, PL spectra from BP3T crystals with DFB systems were narrower than from un-etched crystal, and a clear angular dependence was observed, providing us with the opportunity to evaluate the grating structure in detail. In contrast to Fig. 2e, the resonance peak in Fig. 3a was a single peak, eliminating the possibility of peak splitting by a photonic band gap and suggesting that the effect of the Bragg scattering is caused by difficulties in running experiments from the normal direction. Additionally, we observed two resonant peaks at wavelengths of 565 and 706 nm. Assignment of these peaks is critical in designing superior resonators for laser applications. As a first step, we attempted to determine the waveguide mode using polarized PL measurements. In 1D diffractive gratings, light propagates parallel to the grating vector, and the polarization direction is dependent on the guiding mode (transverse magnetic (TM) or transverse electric (TE)). Due to this relationship, the PL polarization direction is parallel to the grating vector in TM mode. The polarization direction is reversed in TE mode. As shown in Fig. 3c, the guiding mode in our BP3T single crystal with 1D diffractive grating was assigned to the TM mode because polarized PL emission was observed parallel to the grating vector. The TM mode is also reasonable because transition dipole moments in BP3T thin crystals are perpendicular to the crystal surface^23^.

Because lower-order TM modes are superior to higher-order TM modes as resonators, we next attempted to determine the order of each TM mode at wavelengths of 565 and 706 nm. For this analysis, we used the following simple equation to establish the 1D periodic structure: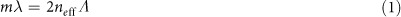 where *m* is the Bragg diffraction resonated order; *λ* is the propagating light wavelength; *n*_eff_ is the effective refractive index of the grating layer; and *Λ* is the grating period^2^. From the PL spectra at 0° (Fig. 3b) and AFM observations (Fig. 1b), *λ* and *Λ* were experimentally determined to be 560 and 350 nm, respectively. Because the light emission that is normal to grating surface is cancelled out when *m* is odd^33^, the observed surface emission indicates that *m* is even (Fig. 1c).

Moreover, *n*_eff_ can be calculated for each TM mode on the basis of the crystal thickness, grating depth, grating structure, and anisotropic refractive indices of the BP3T crystal^36^,^37^,^38^. Because the grating structure of BP3T is a dielectric composite that consists of BP3T line, space, and air regions, the average refractive index of the grating layer *n*_*eff*_ can be estimated by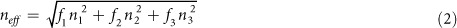 where *f*_*1*_, *f*_*2*_, and *f*_*3*_ are the volume fractions, and *n*_*1*_, *n*_*2*_, and *n*_*3*_ are the refractive indices of the BP3T line, space, and air regions, respectively (*n*_*3*_ = 1)^37^. From the AFM height profile of BP3T single crystals with a 350-nm-period grating, volume fractions were determined to be *f*_*1*_ = 0.51, *f*_*2*_ = 0.25, and *f*_*3*_ = 0.24 (Fig. 1b and 3d).

In addition, we estimated that the refractive index of BP3T crystals from the emission spectra’s interference modulation, which is caused by a pair of parallel crystal edges^36^,^37^. Figure 3e presents the refractive index dispersion of BP3T single crystals. The refractive indices of BP3T single crystals at 565 and 706 nm are 2.60 and 2.30, respectively. Based on these refractive indices of BP3T single crystals, we calculated *n*_*1*_ and *n*_*2*_ with a three-layer planar waveguide model (air/BP3T/SiO_2_) using refractive indices of air (*n*_*air*_ = 1) and SiO_2_ (*n*_*SiO2*_ = 1.47). The effective refractive indices depend on the material thicknesses as well as the thicknesses of the line and space regions of the BP3T grating, which were 363 and 185 nm, respectively.

Profiles of the effective refractive indices were calculated using the eigenmode expansion method with electromagnetic wave analysis software (camfr). Table 1 presents the calculated refractive indices and DFB resonating mode *m* for each TM mode order. From this table and equation (1), we can uniquely determine the resonating mode (*m* = 2) and assign the two resonant peaks at 565 and 706 nm as TM_1_ and TM_0_, respectively. To verify our assignments, we calculated the predicted angular dependence and compared it with experimental results (Fig. 3f), in which the observation is qualitatively well explained by the prediction. The quantitative difference between observation and prediction might be caused by the volume fraction *f*, which is estimated from the AFM height profile. However, as shown in Fig. 3d, our etching accuracy is still insufficient and would lead to larger differences between observation and prediction at higher angles, which is revealed in Fig. 3f.

Because the lower-order feedback of the TM mode is superior to higher-order feedback for stronger light confinement, it is critical for the resonant condition of the TM_0_ mode to be at the top of the PL spectrum peak for electrically driven lasers. However, first-order feedback requires a higher resolution to produce the periodic structure. A 1D grating structure with a periodicity of 270 nm is needed to match the DFB resonant peak to the maximum PL intensity of BP3T single crystals. Our improved UV-NIL method perfectly meets this requirement and successfully demonstrates peak matching between the maximum PL intensity and TM_0_ mode (Supplementary Information S3 and S8).

We should discuss the optoelectrical device parameters of the patterned BP3T SCLETs, which include external quantum efficiency (EQE) and maximum brightness. As shown in the PL emission, EL spectra also revealed strong angular dependency, and without knowing the detail of the angular dependence of the EL emission, we cannot derive the EQE of BP3T SCLETs using the DFB system. However, it is very difficult to collect angular dependent EL spectra under constant voltage applications. To solve this problem, we applied the results of angular dependence in the PL spectra to the analysis of EL optoelectrical parameters. We thereby obtained a maximum brightness of 1.13 × 10^−8^ W and an EL EQE of 1.72 × 10^−3^%. Although the obtained EL EQE is much smaller than the single-crystal PL EQE (13%), our result is approximately one or two orders higher than in a previous report using similar materials, in which the reported EQEs in SCLET with and without the grating structure were 4.9 × 10^−5^% and 3.7 × 10^−4^%, respectively^18^.

**Figure 4:**
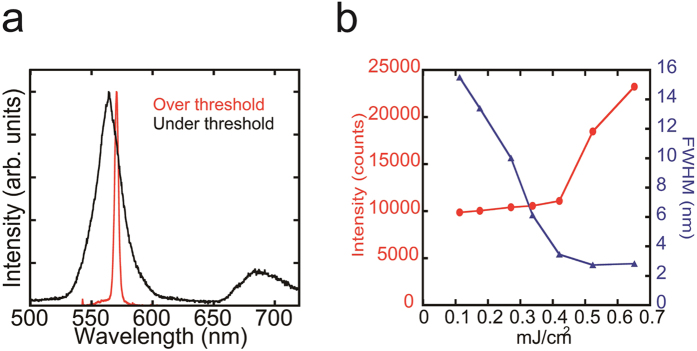
Single-mode lasing in BP3T single crystals with a DFB resonator. (**a**) PL spectra from a BP3T single-crystal surface with a 1D grating structure (350-nm pitch). Black and red lines correspond to excitation under and over the lasing threshold, respectively. (**b**) Dependence of PL integrated intensity and full width at half maximum (FWHM) on pump fluence.

To investigate the potential of BP3T crystals as electrically driven organic semiconductor lasers, the lasing properties of patterned BP3T crystals were examined through high-energy optical pumping. To match the BP3T single-crystal absorption band edge^13^, we selected a pump laser with a wavelength of 481 nm. Emission from the grating feedback structure was detected from the substrate’s surface. As shown in Fig. 4a, we observed single-peak emission under strong excitation, which can be attributed to the TM_1_ mode. Moreover, both the integrated intensity and emission line width exhibited threshold behavior (Fig. 4b), providing solid evidence of single-mode lasing.

The estimated lasing threshold from Fig. 4b is 407 μJ/cm^2^, which is slightly smaller than the previously reported threshold of amplified spontaneous emission (ASE) in BP3T single crystals without a patterning process (520 μJ/cm^2^)^13^, suggesting that the optical gain due to the DFB system is not significant. Although one of the possible origins may be the effect of etching damage, this origin can be ruled out by comparing the ASE thresholds between single crystals with and without etching (Supplementary Information S9), which indicates that insufficient processing accuracy might be the reason for the relatively large lasing threshold.

Finally, we estimated the necessary current density for electrically pumped lasing from the optical threshold and compared it with our achievable current density. We obtained the lowest lasing threshold for a BP3T single crystal with a DFB grating of 407 μJ/cm^2^. This corresponds to a photon density of 9.8 × 10^14^/cm^2^ in the pump laser (excitation laser wavelength of 481 nm). Because the absorption coefficient of BP3T single crystals at 481 nm and the thickness of etched single crystals are 0.052 /μm^39^ and 363 nm, respectively, the excitation density at the lasing threshold (*N*_*th*_) is approximately 5.1 × 10^17^ /cm^3^. This is the minimum exciton density necessary for lasing and must be generated electrically (by EL) to reach the threshold for electrically pumped laser. Importantly, our laser pulse (800 ps) is much shorter than the single exciton life time in the BP3T crystal (*τ* = 1.68 ns)^22^. Assuming that there are no other optical losses, e.g., from carrier-induced absorption, exciton-exciton annihilation and light losses during propagation inside single crystals, then the lasing threshold for the injection current can be estimated by the following equation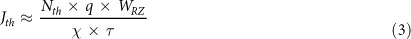 where *q* is the electrical charge, *W*_*RZ*_ is the width of the recombination zone (typically 3 μm in BP3T SCLETs)^40^, and *χ* is the formation ratio of the singlet exciton (0.25). Moreover, the threshold current density is strongly dependent on the thickness of charge accumulation layer. To calculate the current densities, we assumed an accumulation layer thickness of 1 ML for the BP3T single crystal (3 nm) because it has been reported that carrier conduction occurs in only one or two molecular layers at the organic/dielectric interface^10^. Using the accumulation layer thickness, we estimated that the necessary current density is approximately 58 kA/cm^2^, which is 4 times as high as the maximum current density in BP3T SCLETs with the DFB grating (15 kA/cm^2^) and twice as high as the reported highest value among ambipolar BP3T SCLETs (33 kA/cm^2^)^10^. Note that the estimated necessary current density is a minimum estimation because we did not take into account any optical losses, such as carrier-induced absorption, exciton-exciton annihilation and light losses during propagation inside a single crystal. However, as we discussed above, there is still plenty of room for improvement through optimizing the fabrication process, which leads to a decrease in necessary current density for lasing and an increase in the achievable maximum current density in SCLETs.

In summary, we fabricated 1D and 2D grating structures with UV-NIL and dry-etching methods. We observed clear resonant effects that are caused by the 1D grating structure, and more importantly, single-mode lasing was performed under strong photo excitation. Moreover, we succeeded in fabricating ambipolar SCLETs with DFB resonators. These results strongly indicate that the fabrication of photonic crystals with BP3T single crystals is a promising approach to creating new types of organic lighting and lasing devices because of their high refractive index,^37^ high luminescent efficiency,^22^ and high carrier mobility.^23^ The robustness of an organic crystal typified by B3PT underpins these approaches.

## Methods

### Crystal Growth and Substrate Preparation

Single crystals of BP3T were grown by physical vapor transport under an Ar gas stream, which was developed by Bisri *et al.*^23^ A highly doped silicon wafer with a 500-nm thermally oxidized SiO_2_ layer was spin-coated with poly(methyl methacrylate) (PMMA, Sigma Aldrich, St. Louis, Missouri, United States, CO., average Mw ~120,000). The substrate was annealed at 100 °C for 24 h under a N_2_ atmosphere.

### UV-nanoimprint lithography (NIL)

UV-NIL is schematically shown in Fig. 1a and Fig. S1. A Si master mold was patterned by e-beam lithography. We fabricated poly(dimethylsiloxane) (PDMS) molds by pouring a mixture of PDMS precursor and curing agent (10:1 by weight) into the Si master mold. We used SIM-260 and CAT-260 as the PDMS precursor and curing agent, respectively, which were provided by Shin-Etsu Chemical Co., Ltd., Chiyoda, Tokyo, Japan. After thermal curing of the mixture at 70 °C for 1 h and 150 °C for 2 h, the PDMS mold was separated from the master mold. We also spin-coated UV-curable resist (NICT-82) provided by the Daicel Corp., Osaka, Osaka, Japan. (50 w % in 2-methoxy-1-methylethyl acetate) at a speed of 500 rpm for 5 s and 2,000 rpm for 10 s onto the SiO_2_/Si substrate with BP3T crystal. The thickness of the UV-curable resist film was approximately 1.5 μm, which was higher than that of BP3T single crystals (<<1 μm). The typical UV irradiation time was 40 min under a N_2_ atmosphere at ambient pressure. After rest curing, the sample was carefully separated from the PDMS mold. Finally, the UV-curable resist was etched by a SAMCO, Kyoto, Kyoto, Japan. 200iP Inductively Coupled Plasma (ICP) Etching System using Ar gas for 20 min. The ICP power and bias power were set to 90 and 25 W, respectively, and the Ar flow rate was 22 sccm. The process chamber pressure was maintained at 2.0 Pa. The etching rate of the organic single crystal and the UV-curable resist were approximately 40 and 60 nm/min, respectively. The surface properties were measured by atomic force microscope (Bruker Corp., Billerica, Massachusetts, United States, Multimode 8).

### SCLET fabrication

We fabricated SCLETs with the method described by Sawabe *et al.*^10^ The structure of the SCLET with a 1D-grating device is given in Fig. 2a,b. We laminated BP3T single crystals onto the PMMA layer to reduce electron traps and then introduced grating structures to the BP3T single crystal with UV-NIL. Au and Ca asymmetric electrodes were used to promote hole and electron injections. We considered energy level matching between the metal electrode work function and BP3T molecular levels, such as the highest-occupied molecular orbital (HOMO) and lowest-unoccupied molecular orbital (LUMO) levels. We also used cesium fluoride (CsF) thin films (thickness ~ 0.6 nm) as an electron-injection buffer layer. Electrical characterization of the device was performed inside a dark glove box using a semiconducting parameter analyzer (Agilent Technologies, Santa Clara, California, United States, B1505A).

### Optical measurements of BP3T single crystals

EL and PL spectra were collected through an optical microscope (Keyence Corp., Osaka, Osaka, Japan, VH-Z100R) by spectrograph (Newport Corp., Irvine, California, United States, FIC-model 77440) with a Peltier-cooled detector (Andor Technology, Belfast, Contae Aontroma, Northern Ireland, DV401A-UVB). The angular dependence of the PL spectra was also collected by a rotatable optical microscope (Keyence Corp., Osaka, Osaka, Japan, VH-Z100R). Polarized PL spectra were measured through a polarizer that was mounted on an optical microscope (Olympus Corp., Shinjuku, Tokyo, Japan, BX51 M) and were collected by a Shamrock SR303i (Andor Technology, Belfast, Contae Aontroma, Northern Ireland). The photo-pumped laser spectra were collected by irradiating the BP3T samples with a pulsed laser beam and the lasing characteristics were observed normal direction to the crystal surface. The pump pulses were generated by a dye laser (Coumarin 481, Exciton Corp., Dayton, Ohino, United States) that was excited by a nitrogen gas laser (Optical Building Blocks Corp., Edison, New Jersey, United States, GL-3300 λ = 337 nm with a pulse width of 1ns). The pump pulses were generated at a wavelength of λ = 481 nm, chosen to match the BP3T absorption band edge, and a pulse width of 800 ps. A neutral density filter was used to adjust the excitation intensities. The laser beam was focused on BP3T crystals with a size of 0.52 mm^2^ and an incident angle of 45 degree. The photo-pumped DFB laser emission from a BP3T single crystal was collected through the microscope (Nikon Corp., Chiyoda, Tokyo, Japan, eclipse) with a multichannel spectrograph (Lambda Vision Inc., Sagamihara, Kanagawa, Japan, SA-100C-HPCB/C). The photo-pumped laser measurements were performed under atmospheric conditions at room temperature.

## Author Contributions

T.T., K.M., and K.S. conceived and designed the experiments. S.H. provided the BP3T powder. K.M., J.L., and W.T. performed the optical measurements and data analysis. K.M. and K.S. fabricated the grating structures and SCLETs. Y.I. and T.S. contributed to the interpretation of results. T.T., K.M., and T.S. wrote the manuscript. All authors discussed the results and commented on the manuscript.

## Additional Information

**How to cite this article**: Maruyama, K. *et al.* Ambipolar light-emitting organic single-crystal transistors with a grating resonator. *Sci. Rep.* **5**, 10221; doi: 10.1038/srep10221 (2015).

## Supplementary Material

Supplementary Information

Supplementary Information

## Figures and Tables

**Table 1:** Summary of effective refractive indices and DFB resonating mode.

	***n***_***1***_	***n***_***2***_	***n***_***eff***_	***m***
*λ* = 565 nm				
TM_0_	2.5	2.2	2.1	2.6
TM_1_	2.1	1.4	1.7	2.1
TM_2_	1.5	1.2	1.3	1.6

*λ* = 706 nm				
TM_0_	2.2	1.8	1.9	1.9
TM_1_	1.8	1.4	1.5	1.5
TM_2_	1.4	1.2	1.3	1.3

## References

[b1] Forrest S. R. The path to ubiquitous and low-cost organic electronic appliances on plastic. Nature 428, 911–918 (2004). 15118718 10.1038/nature02498

[b2] Samuel I. D. W. & Turnbull G. A. Organic semiconductor lasers. Chem. Rev. 107, 1272–1295 (2007). 17385928 10.1021/cr050152i

[b3] Nakanotani H. *et al.* Highly balanced ambipolar mobilities with intense electroluminescence in field-effect transistors based on organic single crystal oligo(p-phenylenevinylene) derivatives. Appl. Phys. Lett. 95, 033308 (2009).

[b4] Nakanotani H. *et al.* Emission Color Tuning in Ambipolar Organic Singe-Crystal Field-Effect transistors by Dye-Doping. Adv. Funct. Mater. 20, 1610–1615 (2010).

[b5] Hotta S. & Yamao T. *et al.* The thiophene/phenylene co-oligomers: exotic molecular semiconductors integrating high-performance electronic and optical functionalities. J. Mater. Chem. 21, 1295–1304 (2011).

[b6] Tang C. W. *et al.* Electroluminescence of doped organic thin films. J. Appl. Phys. 65, 3610–3616 (1989).

[b7] Baldo M. A. *et al.* Prospects for electrically pumped organic lasers. Phys. Rev. B: Condens. Matter 66, 035321 (2002).

[b8] Giebink N. C. & Forrest S. R. Quantum efficiency roll-off at high brightness in fluorescent and phosphorescent organic light emitting diodes. Phys. Rev. B: Condens. Matter 77, 235215 (2008).

[b9] So F. *et al.* Organic light-emitting devices for solid-state lighting. MRS Bull. 33, 663–669 (2008).

[b10] Sawabe K. *et al.* Current-Confinement Structure and Extremely High Current Density in Organic Light-Emitting Transistors. Adv. Mater. 24, 6141–6146 (2012). 22961877 10.1002/adma.201202252

[b11] Ichikawa M. *et al.* Photopumped laser oscillation and charge-injected luminescence form organic semiconductor single crystals of a thiophene/phenylene co-oligomer. Appl. Phys. Lett. 87, 221113 (2005).

[b12] Mizuno H. *et al.* Single Crystals of 5,5’-Bis(4’-methoxybiphenyl-4-yl)-2,2’-bithiophene for Organic Laser Media. Adv. Mater. 24, 5744–5749 (2012). 22930494 10.1002/adma.201202470

[b13] Bisri S. Z. *et al.* Organic Single-Crystal Light-Emitting Transistor Coupling with Optical Feedback Resonators. Sci. Rep. 2, 985 (2012). 23248748 10.1038/srep00985 PMC3523286

[b14] Fujiwara S. *et al.* Laser oscillations of whispering gallery modes in thiophene/phenylene co-oligomer microrings. Appl. Phys. Lett. 91, 021104 (2007).

[b15] Sasaki F. *et al.* Microdisk and Microring Lasers of Thiophene-Phenylene Co-oligomers Embedded in Si/SiO_2_ Substrates. Adv. Mater. 19, 3653–3655 (2007).

[b16] Sasaki F. *et al.* Microdisk lasers and field effect transistors of thiophene/phenylene co-oligomers by using high temperature deposition method. Org. Electron. 11, 1192–1198 (2010).

[b17] Fang H.-H. *et al.* Distributed Feedback Lasers Based on Thiophene/phenylene Co-Oligomer Single Crystals. Adv. Funct. Mater. 22, 33–38 (2012).

[b18] Yamao T. *et al.* Current-Injected Spectrally-Narrowed Emissions from an Organic Transistor. Adv. Mater. 22, 3708–3712 (2010). 20518039 10.1002/adma.201000171

[b19] Makino Y. *et al.* Spectrally-Narrowed emissions from Organic Transistors Composed of Layered Crystals Laminated on a Two-Dimensional Diffraction Grating (Spectral Narrowing from Organic Crystal Transistors). Mol. Cryst. Liq. Cryst. 566, 8–12 (2012).

[b20] Okada A. *et al.* Current-injected narrow linewidth emissions from organic-crystal light-emitting transistors having a diffraction grating. Phys. Status Solidi C 9, 2545–2548 (2012).

[b21] Ding R. *et al.* Distributed feedback lasing from thin organic crystal based on active waveguide grating structures. Org. Electron. 13, 1602–1605 (2012).

[b22] Kanazawa S. *et al.* Self-waveguided photoemission and lasing of organic crystalline wires obtained by an improved epitaxial growth method. Chem. Phys. Chem. 7, 1881–1884. (2006). 16952121 10.1002/cphc.200500669

[b23] Bisri S. Z. *et al.* High Mobility and Luminescent Efficiency in Organic Single-Crystal Light-Emitting Transistors. Adv. Funct. Mater. 19, 1728–1735 (2009).

[b24] Yamao T. *et al.* Direct Formation of Thin Single Crystals of Organic Semiconductors onto a Substrate. Chem. Mater. 19, 3748–3753 (2007).

[b25] Gates B. D. *et al.* New Approaches to Nanofabrication: Molding, Printing, and Other Techniques. Chem. Rev. 105, 1171–1196 (2005). 15826012 10.1021/cr030076o

[b26] Schift H. Nanoimprint lithography: An old story in modern times? A review. J. Vac. Sci. Technol. B. 26, 458–480 (2008).

[b27] Maruyama K. *et al.* Fabrication of one-dimensional grating structure on organic single-crystal surface. Jpn. J. Appl. Phys. 53, 02 MC19 (2014).

[b28] Hotta S. *et al.* Synthesis of thiophene/phenylene co-oligomers. II[1]. block and alternating co-oligomers. J. Heterocycl. Chem. 37, 281–286 (2000).

[b29] Chua L.-L. *et al.* General observation of n-type field effect behaviour in organic semiconductors. Nature 434, 194–199 (2005). 15758994 10.1038/nature03376

[b30] Zaumseil J. *et al.* Spatial control of the recombination zone in an ambipolar light-emitting organic transistor. Nat. Matter 5, 69–74 (2006).

[b31] Swensen J. S. *et al.* Light emission from an ambipolar semiconducting polymer field-effect transistor. Appl. Phys. Lett. 87, 253511 (2005).

[b32] Takahashi T. *et al.* Ambipolar Light-Emitting Transistors of a Tetracene Single Crystal. Adv. Funct. Mater. 17, 1623–1628 (2007).

[b33] Hunsperger R. G. Integrated Optics: Theory and Technology 3rd edn (Springer-Verlag, Berlin, 1991).

[b34] Riechel S. *et al.* Laser modes in organic solid-state distributed feedback lasers. Appl. Phys. B. 71, 897–900 (2000).

[b35] Turnbull G. A. *et al.* Relationship between photonic band structure and emission characteristics of a polymer distributed feedback laser. Phys. Rev. B. 64, 125122 (2001).

[b36] Yamao T. *et al.* Dispersion of the refractive indices of thiophene/phenylene co-oligomer single crystals. J. Appl. Phys. 110, 053113 (2011).

[b37] Takahashi W. *et al.* Optical characteristic of 5,5”-bis(4-biphenylyl)-2,2’:5’,2”terhiophene single-crystal thin-film resonator. Jpn. J. Appl. Phys. 53, 02BB02 (2014).

[b38] Matsuu M. *et al.* Formation of Periodically Ordered Zinc Oxide Nanopillars in Aqueous Solution: An Approach to Photonic Crystals at Visible Wavelengths. Adv. Mater. 18, 1617–1621 (2006).

[b39] Bisri S. Light-Emitting towards Current-Induced Amplified Spontaneous Emission in Organic Single Crystals. *PhD Thesis*, Tohoku University (2011).

[b40] Bisri S. *et al.* p-i-n Homojunction in Organic Light-Emitting Transistors. Adv. Mater. 23, 2753–2758 (2011). 21608046 10.1002/adma.201004572

