# Supplementary material for: Ambipolar light-emitting organic single-crystal transistors with a grating resonator
Source: Sci Rep. 2015 May 11;5:10221. doi: 10.1038/srep10221 (PMC4426699; doi:10.1038/srep10221)
Supplement: Supplementary Information [file srep10221-s1.pdf]

Supporting Information for

Ambipolar light-emitting organic single-crystal transistors with a grating resonator

Kenichi Maruyama[1], Kosuke Sawabe[2], Tomo Sakanoue[1], Jinpeng Li[1], Wataru Takahashi[1], Shu Hotta[3], Yoshihiro Iwasa[4], & Taishi Takenobu[1]*

[1]Department of Applied Physics
Graduate School of Advanced Science and Engineering
Waseda University
Tokyo 169-8555, Japan
E-mail: takenobu@waseda.jp

[2]Department of Physics
Graduate School of Science
Tohoku University
Sendai 980-8578, Japan

[3]Department of Macromolecular Science and Engineering
Kyoto Institute of Technology
Kyoto 606-8585, Japan

[4]Quantum-Phase Electronics Center and Department of Applied Physics
The University of Tokyo
Tokyo 113-8656, Japan
RIKEN Center for Emergent Matter Science (CEMS), Wako 351-0198, Japan

Keywords: transistors, photonics, organic electronics, ambipolar semiconductors, resonators

S1. Fabrication methods

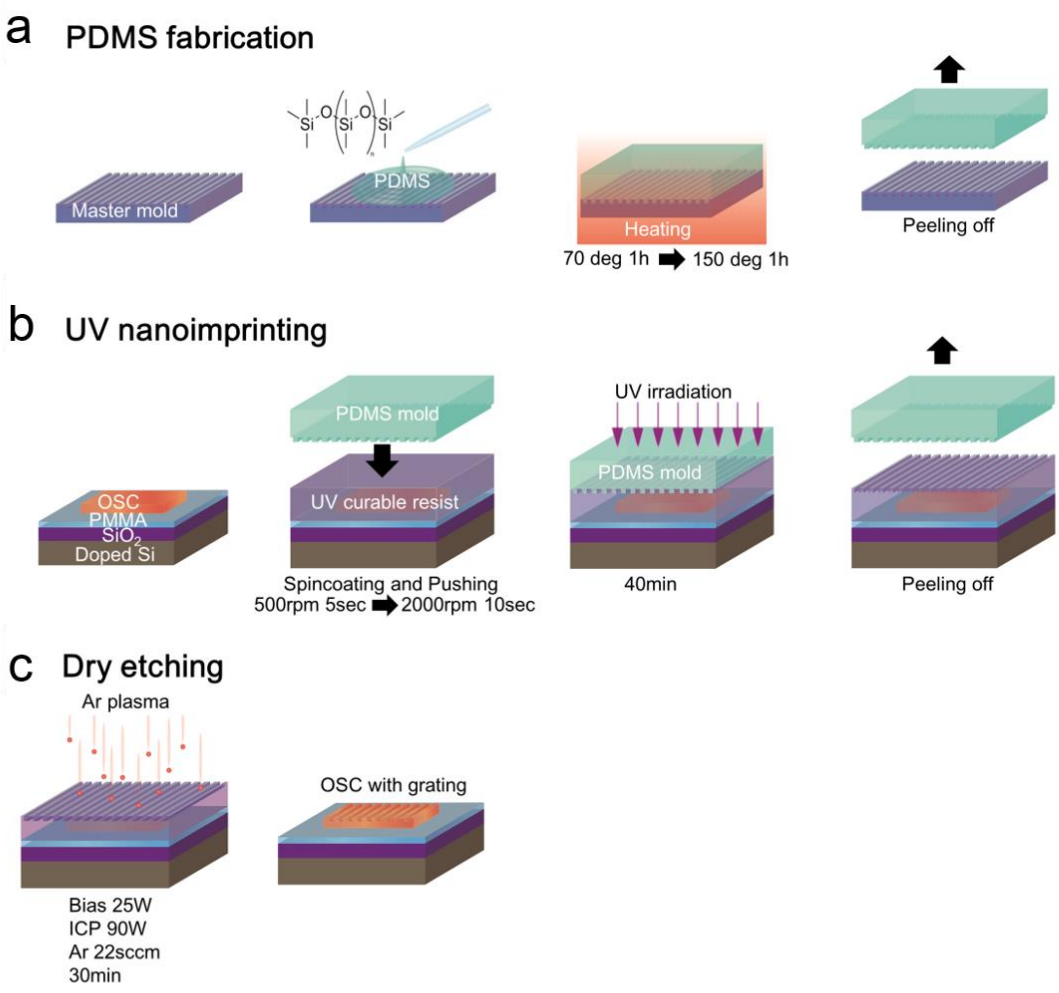

Figure S1 | Schematic representation of the fabrication methods used.

S2. Optimization of the NIL and dry etching methods

PDMS peeling-off conditions. As shown in Fig. S2a, the quality of the Si master mold is extremely high, and its periodicity is designed to have a 350-nm pitch. However, AFM images of UV-curable resist films after nano-imprinting (Fig. S2b and S2c) were strongly sample dependent, so modification of the method was necessary. We observed two important points: i) The direction of PDMS peeling-off should be perpendicular to the grating vector, and ii) the peeling-off speed should be less than 1 mm/s. With these two optimizations, we reproducibly obtained grating structures similar to Fig. S2b.

UV exposure time, dry etching power, and etching gas species. UV exposure time, dry etching power, and etching gas species were observed to be extremely important for fabricating fine structures on BP3T crystals. After many experiments, we obtained good fabrication conditions (UV exposure time of 1 h, etching bias power of 25 W, and Ar plasma) and demonstrated excellent pattern formation (Fig. S2d). Without this optimization, AFM images after dry etching exhibited poor pattern formation, indicating the importance of these parameters (UV exposure time of 10 min (Fig. S2e), bias power of 35 W (Fig. S2f), and He plasma (Fig. S2 g)).

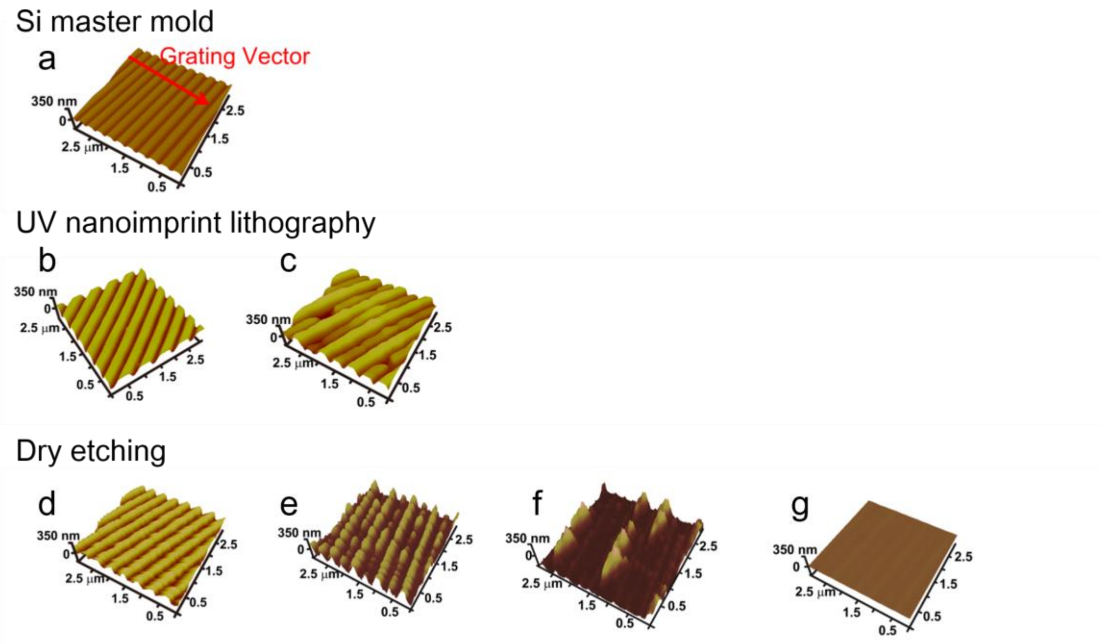

Figure S2 | Optimization of UV-NIL methods. AFM images of the Si master mold (**a**), UV-curable resists after NIL (**b**, **c**), and the surface of the etched BP3T crystal (**d**, **e**, **f**, **g**). The peeling-off speeds are (**b**) less than 1 mm/s and (**c**) more than 1 mm/s. (**d**) BP3T single crystals after dry etching. The UV exposure time is 1 h, the bias power is 25 W, and Ar gas is used. (**d**), (**e**), (**f**), and (**g**) present AFM images of samples after dry etching with different conditions. (**e**), (**f**), and (**g**) correspond to a different UV irradiation time (10 min.), bias power (35 W), and etching gas (He gas), respectively.

S3. AFM images of etched BP3T single crystals

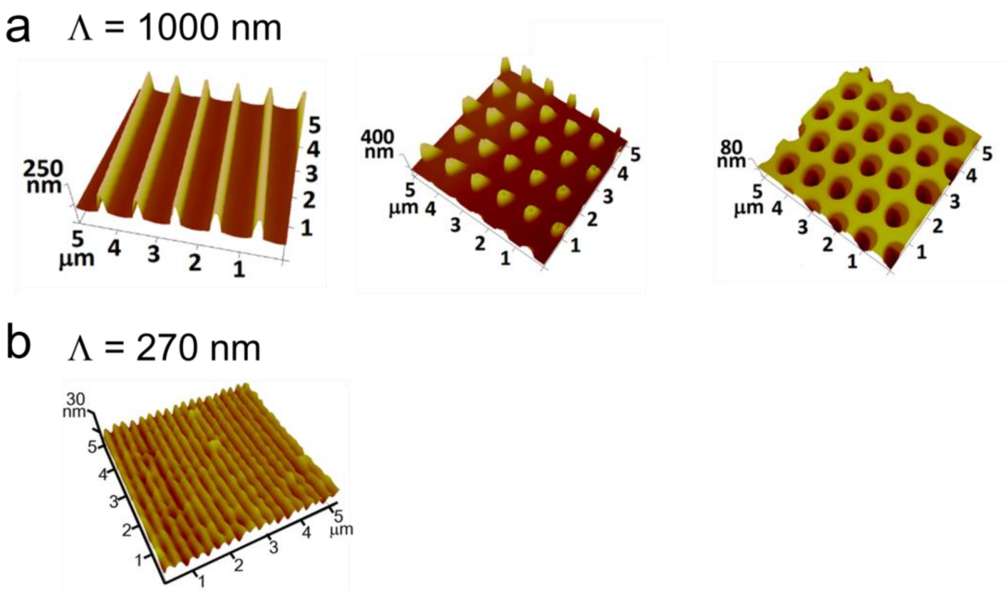

Figure S3 | AFM images of etched BP3T single crystals. (**a**) The grating periods are 1,000 nm wide. The AFM images present 1D, 2D pillar, and 2D hole structures. (**b**) 1D grating structure with a 270-nm period.

S4. Effect of UV exposure on the SCLET characteristics

The UV-NIL method requires UV exposure to cure the UV resist. As we explained in Supplementary Information S2, a longer UV irradiation time was better for high-quality patterning. Therefore, we started with a very long irradiation time (2 h). However, the electron current of the SCLET obtained with long irradiation times was extremely small, and we could not demonstrate strong light emission (Fig. S4a).

We tried to find the dominant parameter for this negative effect and successfully improved the electron current by reducing the irradiation time from 2 h to 40 min (Fig. S4b).

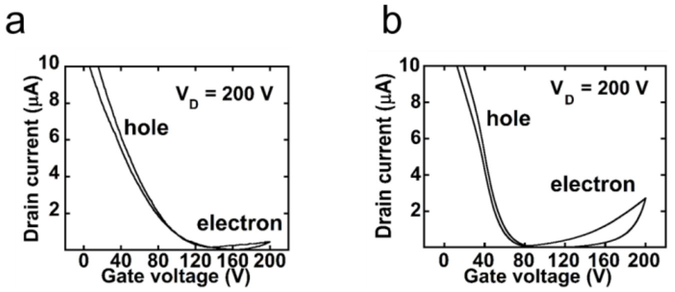

Figure S4 | Effect of UV exposure time on the SCLET transfer characteristics. (**a**) and (**b**) show the transfer characteristics of SCLETs with UV exposure times of 2 h and 40 min, respectively.

S5. EL image of BP3T SCLET with a 350-nm period grating structure

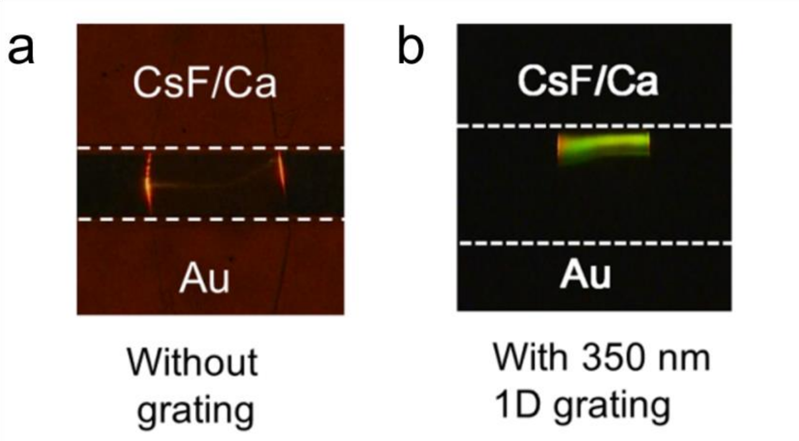

Figure S5 | EL images of a BP3T SCLET. (**a**) Without a grating structure. (**b**) With a 350-nm period grating structure.

S6. Device statistics of fabricated BP3T SCLETs with DFB grating

Table S6 | Device statistics of fabricated BP3T SCLETs with DFB grating.

	Device type	Hole mobility (cm^2/Vs)	electron mobility (cm^2/Vs)	Hole Vth (V)	Electron Vth (V)
Device #1	ambipolar	2.1	0.014	-131	52.5
Device #2	ambipolar	1.56	0.00353	-125	32.4
Device #3	unipolar	Not calculated	No current		
Device #4	unipolar	Not calculated	No current		
Device #5	ambipolar	1.17	0.0245	-101	90.6
Device #6	ambipolar	1.07	0.049877	-135	66.49

S7. Device comparison between patterned BP3T SCLETs and BP3T SCLETs without etching

Table S7 | Device comparison between patterned BP3T SCLETs and BP3T SCLETs without etching.

	Ref [23]	Ref [13]	Ref [10]	BP3T LET with grating (this work)
Hole mobility (cm^2/Vs)	1.64	1.0	0.2	2.1
electron mobility (cm^2/Vs)	0.17	0.2	0.2	0.014
Hole Vth (V)	-	-	-50	-131
Electron Vth (V)	160	-	50	52.5

S8. PL spectra of a BP3T single crystal with a 270-nm grating period

Figure S8 presents the spectra detected normal to the waveguide plane of BP3T single crystals with and without 270-nm gratings. The presence of the grating structure (modulation depth of 30 nm, period of 270 nm) controls PL emission. From a simple calculation using equation (1), the effective index, waveguide mode, and DFB mode were determined to be $n_{eff} = 2.2$, TM_0 and $m = 2$, respectively, indicating excellent matching between the PL peak and resonant wavelength.

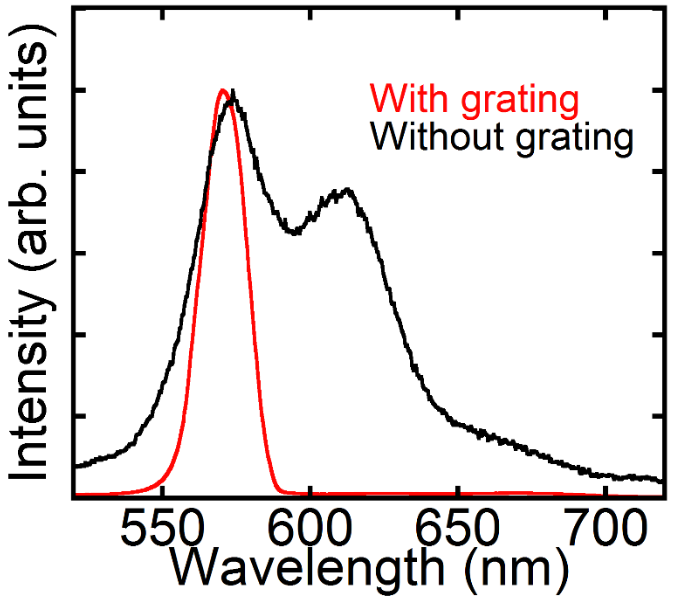

Figure S8 | PL spectrum of a BP3T single crystal with a 270-nm period (red) and without a grating structure (black). The peak wavelength is at 570 nm.

S9. Effect of etching process

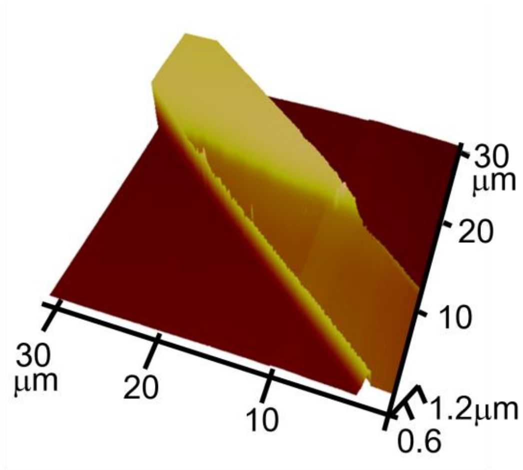

Figure S9 | AFM image of a BP3T single crystal after the etching process. The bright area was covered by a metal mask during the etching process.

Table S9 | Effect of the etching process.

	Rms (nm)	Ra (nm)	ASE threshold (uJ/cm^2)
Before etching	1.26	18.2	3778
After etching	1.26	15.5	2472
